# Cases of Impacted Cæcum and Colon

**Published:** 1879-12-20

**Authors:** E. R. Maxson

**Affiliations:** Syracuse, New York


### *CASES OF IMPACTED CÆCUM AND COLON.*

BY E. R. MAXSON, M.D., L.L.D.,
of Syracuse, New York.

As most of the cases of this character, of long standing, to which I have been called, had been regarded and treated as other affections, I offer the following, which have fallen under my treatment during the past two years, hoping to throw some light upon the subject:

June 28th, 1878, I was called to attend a boy about nine years old. of good parentage, who was suffering from tuberculous meningitis, as was evident from all the symptoms of that disease being fully developed, but which I need not enumerate.

On inquiring into the cause, I found that from infancy he had been badly constipated, and for years had lumps in his bowels which had been regarded as "tumors" of some kind, not defined.

He had, from infancy, suffered much from pain in his head, at times severe, and for several months before I saw him it had been intense, rendering him by turns delirious.

I discovered that the *cœcum* and *colon* were impacted, constituting the "tumors," and that the condition had been of long standing, evidently impairing digestion, and affecting the brain sympathetically, doubtless leading to the tuberculous meningeal condition in which I found him.

My prognosis was unfavorable, of course; even after having removed—by rhubarb and Epsom salts, with injections of warm water, containing salt, lard, molasses and beef's gall, of each a teaspoonful to the half pint—an enormous quantity of hardened scybala, entirely dispersing the abdominal tumors; for, though it gave him great temporary relief, as I anticipated and warned the parents, the tuberculous symptoms eventually passed on to a fatal termination.

October 24th, 1877, I was called to attend a highly respectable gentleman about fifty years old, of a fair constitution and excellent habits, who had been in poor health for nearly ten years; having had little appetite, imperfect digestion, a fistula in ano, some cough, and during the entire period what is called a "hardened stomach," his mother having died of a similar affection.

He had been under treatment more or less of the ten years, steadily growing worse up to the time I saw him, when his case had been declared hopeless, mainly from his lungs having become tuberculous, though no matter had yet been expectorated. as appeared. I discovered that what had been regarded as a hardened stomach was really an impacted colon, as was clearly demonstrated by the effects of gentle laxatives, with electricity, which brought away nearly three feet in length of impacted fæces from the colon, entirely removing the hardness, which having been felt most along the transverse colon, had been called a hardened stomach.

This impacted condition of the large intestine had evidently caused, mechanically and otherwise, the indigestion, loss of appetite, fistula in ano, marasmus, pulmonary vomiciæ, etc. Its removal gave him great relief, and left the lung disease to lead on to a fatal termination.

On post-mortem examination I found a scrofulous-appearing tumor (*not scirrhous*), on the duodenum, about two inches below the stomach, with a congested appearance of this portion or the small intestines, all evidently caused by the mechanical impression of the impacted colon on the part. And I have no doubt but that the fistula in ano was the direct and indirect result of the abnormal accumulation, as is often the case.

I was called January 10th, 1878, by a gentleman to attend his wife, about fifty years old, in the last stages of what had been regarded and treated as an organic disease of the liver, attended with a tumor of that organ. Her great distress, at the time, was enormous swelling and intense pain in her right leg and foot, for which she desired relief.

I found an impacted colon and cæcum, the latter pressing so heavily (now that she was in bed), upon the right iliac vein and lymphatics as to have caused the swelling and pain, the nerves of course being involved, directly and indirectly.

I very much feared that it had pruduced scirrhus of the pyloric extremity of the stomach or duodenum, as the impaction was evidently of long standing. The ureter of that side was evidently obstructed, as there was intense pain in the right lumbar region, with some swelling.

I gave her fluid extract of dandelion, 4 gm. (ʒj), every morning, and fluid extract of rhubarb and Epsom salts, of each, 4 gm. (ʒj), at evening, directing injections of warm water, with salt, lard, molasses and beef's gall, of each a teaspoonful to 240 cc, (℥viij), at evening.

On the third day a large amount of partially softened scybala came away, relieving the right leg and foot of pain and swelling, and the kidney somewhat.

But simultaneously with this, the partially restrained scybala, lodging in the descending colon, near the sigmoid flexure, caused a similar swelling, with pain, in the left leg and foot. But two days later, on removal of this retained matter, this also suddenly subsided, as did a pain and swelling in the left lumbar region, from a partial closure of the ureter by the mechanical pressure being thus relieved.

The tumor, which had been regarded as hepatic, had now subsided, except an induration in the pyloric region. And though the patient was now free from pain and swelling, and quite comfortable, she was little more than skin and bones, and died about a week later.

I made an examination by request of the family, finding, as I had anticipated, *scirrhus* of the pyloric extremity of the stomach and commencement of the duodenum, evidently the result of pressure of the fecal matter in the large intestine passing over it; the kidney of the right side being loosened, congested, and very much enlarged, from pressure upon its excretory duct. The liver was normal, and so were all the other organs, except in so far as they were affected by the general marasmus.

I was called January 26th, 1878, to see a prominent gentleman, about fifty years old, who had been very much constipated for several years, "blood and slime" passing instead of fecal matter, as they informed me.

During the year before I saw him he had run down, suffering the most intolerable tenesmus, and disposition for, without much, movement of his bowels; it having been necessary to draw his water, as I learned, a part of the time.

For twelve weeks before I saw him he had been confined to the house, and at last, more or less to his bed, his case having been regarded and treated, I believe, as dysentery. I did not find positive evidence of any abdominal tumor in this case, as the abdomen was so much bloated it was impossible to diagnose the case by palpitation; but suspecting fecal impaction of the colon, I passed my finger into the rectum and found its veins nearly the size of my little finger, and also rectal ulceration.

My diagnosis was an impacted colon. And, with proper remedies, as in the other cases, I succeeded, within two weeks, in bringing away about half a bushel of intensely hardened scybala, which had evidently interrupted micturition, led to the intolerable tenesmus daily, caused the hemorrhoidal congestion and ulceration, and brought him to death's door, as it were, for all these unpleasant complications subsided with the removal of the retained matter, except the rectal ulceration, which was cured by touching, through a speculum, with nitrate of silver. In six weeks he was able to resume his accustomed arduous and responsible business, which he has pursued, with increasing flesh and strength, to the present time, I believe.

An invalid gentleman, forty-five years old, called on me May 13th, 1878, from a distant city, of which he is a resident, to consult me in relation to an abdominal tumor that had been diagnosed as a "loose kidney," as I learned from him, by many prominent physicians, it having been, however, previously poulticed for a long time, preparatory to opening as an abscess. The tumor occupied the right anterior abdominal region, being about the size of two fists, I think. I found that he had been badly constipated for a long time before he discovered the tumor, which had been sore or tender and painful at times, now rendering him unable to work. My diagnosis was impacted scybala at the junction of the ascending and transverse colon, with possibly an intestinal tumor, as an effect of it, benign or malignant, but probably scrofulous. And with this view, I treated him on the principles already laid down, with laxatives, tonics, etc. On May 31st hardened scybala passed him, leaving the tumor about two-thirds its previous size. And again, June 14th, still more passed, reducing it to about one-third, so that he threw off his bandage or belt, which he had been obliged to wear, and under a laxative and tonic treatment rapidly regained his flesh and strength, enabling him to resume his responsible and arduous labors, which he continued until last August, when he again lost flesh and strength, having suspended treatment, and as I have since learned, by the advice of prominent physicians, who regarded the tumor as a loose kidney; he was without treatment through the winter and early spring of 1878 and 1879, except morphine, to quiet pain which was intense, as I learned, from April 1st till I saw him, May 17th, 1879, when he was very low. Feeling confident that the tumor was connected with the intestine, and probably the result of the impacted colon, and of a scrofulous character, and fully satisfying myself that it had been inflamed, suppurated and then contained matter, notwithstanding the high authority and positive assurance of his medical advisers to the contrary, I resolved at once to let the matter out. This I did by introducing the aspirator needle, when, with a very excellent instrument, I drew out 240 cc. (half a pint) of thick, scrofulous-appearing matter, affording the patient instantaneous and almost perfect relief from the intense pain from which he had suffered for six weeks or longer. I gave him tonics and alteratives, directed a flannel about the abdomen, a solution of iodide of potassium, 8 gm. (ʒij) to the 30 cc. (℥j) of glycerin, to be applied to the abdomen morning and evening, and a small poultice to be kept over the puncture with the hope of keeping it open for the discharge while there was matter.

The matter continued to flow from the puncture for two weeks, and he had improved steadily and rapidly. One week after I drew the matter he was in the garden; and June 13th, being less than a month, he came to see me, a distance of one hundred and thirty miles, and since July 24th—the tumor having diminished in size, and the hectic, colliquative diarrhœa and night sweats having entirely disappeared, and his appetite having become excellent, gaining, from June 18th to July 24th, twenty-nine pounds in weight—he has been engaged regularly in his former occupation, that of a railroad engineer, as I am informed.

The tumor may have been hydatid, and in that case might have caused the impaction of the colon. But from all the symptoms and circumstances and from what I have found in other cases, it was probably the result of the accumulation of scybala in the colon, and of a scrofulous character, having thus inflamed and suppurated.

I never believed it was a loose kidney, as it was not like cases of that kind that I have examined in this country and abroad, one of the most interesting of which I examined with the late Sir James Y. Simpson, in Edinburgh, another in this city, and still another in Geneva, a few years since.

Too much care cannot be taken in diagnosing such cases, as upon this depends the treatment or no treatment, and hence, often, the comfort, health, and even life of the unfortunate patient.

June 1st, 1878, there was brought to this city and placed under my care, a gentleman from a distant part of the State, fifty years old. He had been for a few months in a gloomy mood, and for the previous week insane, and under treatment for it in two of the principal cities of this State and in a distant rural district, from which he was taken to an insane asylum on a certificate of insanity from two of his attendants, of respectability, I believe.

But as no very favorable prognosis was given at the asylum, and as his friends were not pleased with some of the treatment there, the impression having prevailed, as I learned, that his insanity was from organic brain disease, which some of his symptoms might well have been suspected as indicating, he was brought to me.

I learned that insanity was hereditary in the family; that he would not eat; could not sleep much, and that his bowels had moved but once in two weeks.

Regarding this train of facts and symptoms as indicating incipient impaction of the colon, I induced him to take freely pills of aloes and rhubarb, two or three times a day, which soon brought away a large amount of hardened fæces, apparently relieving the brain at once. This treatment, together with pepsin, blood and nerve tonics, with a little cupping in the cervical and lumbar regions of the spine, fully restored him to reason within three weeks.

He gained about twenty pounds of flesh within a month, leaving the city in good health and spirits, July 5th, one month and five days from the time I first saw him, and up to the last account I have had of him there had been no return of his physical or mental derangement.

It is not probable that the impacted colon in this case, had there not been a hereditary pre-disposition, would have produced insanity. But with it, and a slight spinal irritation, it was sufficient; and its removal effected a cure.—*Medical and Surgical Reporter.*

